# Operative Treatment of Facial Neuralgia

**Published:** 1897-08

**Authors:** 


### OPERATIVE TREATMENT OF FACIAL NEURALGIA.

In the *Annals of Surgery*, November, 1896, Dr. McLane Tiffany, of Baltimore, publishes an analysis of one hundred and eight cases of facial neuralgia treated by intracranial operation. Of these nearly two-thirds were subjected to the Hartley-Krause method, and nearly one-fourth to the Rose. Of these cases twenty-four were fatal, including one in which the patient died three months after the operation from cerebral abscess. Shock and sepsis, it is shown, are the chief causes of death. The author, in discussing the result of surgical treatment in the cases of recovery, asserts that intracranial excision of branches of the fifth nerve relieves pain, certainly for a time, and perhaps permanently; but pain may recur, possibly in the terrritory subject to the excised branch, possibly in other branches. Recurrence of pain after known removal of the ganglion of Gasser is not recorded. The expediency of removing the whole of the ganglion is questioned. The first branch, it is stated, is never affected alone.

It is well, in the author's opinion, not to take away the upper portion of the ganglion and the first branch, but rather to remove the second and third branches with the corresponding portion of the ganglion. The following suggestions are made with the object of assisting the surgeon in his decision with regard to the necessity of a central operation in a case of facial neuralgia. An intracranial operation is indicated if more than one branch of the fifth nerve is affected; the painful area receives filaments from the branches near the exit from the head; if the pain is not the expression of a constitutional disorder; if a cause central to the ganglion does not exist; if other measures have failed to give relief. The intracranial operation which should be done is removal of the lower two-thirds of the Gasserian ganglion, together with the second and third branches as far as their foramina of exit from the skull, all in one piece, so as to be certain of the amount of tissue taken away.—*Maryland Medical Journal.*

